# *Adiponectin*-11377CG Gene Polymorphism and Type 2 Diabetes Mellitus in the Chinese Population: A Meta-Analysis of 6425 Subjects

**DOI:** 10.1371/journal.pone.0061153

**Published:** 2013-04-09

**Authors:** Yan-yan Li, Zhi-jian Yang, Chuan-wei Zhou, Xiang-ming Wang, Yun Qian, Jian Xu, Bei Wang, Jun Wu

**Affiliations:** Department of Geriatrics, First Affiliated Hospital of Nanjing Medical University, Nanjing, China; Sapienza University, Italy

## Abstract

**Background:**

Although *adiponectin* −11377CG gene polymorphism is implied to be associated with increased type 2 diabetes mellitus (T2DM) risk, results of individual studies are inconsistent.

**Objective and Methods:**

A meta-analysis consisting of 12 individual studies, including a total of 6425 participants, was carried out in order to investigate the association of *adiponectin* −11377CG gene polymorphism with T2DM. The pooled odds ratio (OR) and its corresponding confidence interval (CI) at 95% were assessed through the random- or fixed- effect model.

**Results:**

A significant relationship was observed between *adiponectin* −11377CG gene polymorphism and T2DM under allelic (OR: 1.150, 95% CI: 1.060 to 1.250, *P* = 0.001), recessive (OR: 1.450, 95% CI: 1.180–1.770, *P* = 0.0004), dominant (OR: 1.071, 95% CI: 1.013–1.131, *P* = 0.015), additive (OR: 1.280, 95% CI: 1.090–1.510, *P* = 0.002), and homozygous genetic models (OR: 1.620, 95% CI: 1.310–1.990, *P*<0.00001). No significant association was found between them under the heterozygous genetic model (OR: 1.640, 95% CI: 0.850–3.170, *P* = 0.140).

**Conclusions:**

*Adiponectin* −11377CG gene polymorphism was significantly associated with T2DM risk susceptibility. G allele carriers are predisposed to T2DM risk.

## Introduction

The epidemic of type 2 diabetes mellitus (T2DM) is considered a major public health problem in China, as it can lead to premature cardiovascular morbidity and death. T2DM is associated with a complicated interaction between genetic mutants and environmental factors. Adipose tissue, as an energy storage depot, is an active endocrine organ that secretes various proteins involved in the regulation of glucose, lipid metabolism, and energy homeostasis [1].

Adiponectin is a specific protein secreted by adipocytes; it participates in the regulation of glucose and lipid metabolism, amelioration of insulin resistance (IR), improvement of the insulin sensitivity, and has anti-inflammation and anti-atherosclerosis effects [2]. Adiponectin is the exclusive adipokine of which the plasma concentration is reduced when the adipose tissue volume is increased [3]. Hypoadiponectinemia has been detected in T2DM or obesity patients [4]. Berg et al. found that adiponectin administration could abolish hyperglycemia in diabetic mice and further suppress the adipocytes to produce glucose [5].

Adiponectin is encoded by one of the most abundant adipose gene transcripts (ADIPOQ), also referred to as the 30 kDa adipocyte complement-related protein (ACRP30) and 28 kDa gelatin binding protein (GBP28). Adiponectin comprises 244 amino acids. The *adiponectin* gene, located in 3q27, spans 16 kb and contains 3 exons and 2 introns. The *adiponectin* rs266729 locus point, −11377CG mutation in the proximal promoter is cytosine (C), which is substituted by guanine (G).

Gu et al. reported that *adiponectin* −11377CG gene polymorphisms contributed to T2DM in Swedish Caucasians [6]. Although a large number of studies on the relationship between *adiponectin* −11377CG gene polymorphisms and T2DM have been carried out domestically, the individual studies results were found to be controversial.Ye et al. (2008) observed that *adiponectin* −11377CG gene polymorphism was associated with the serum adiponectin level, which suggested that this locus polymorphism might increase the T2DM hereditary risk in the Shanxi Chinese population [7]. Analogously, Sun et al. (2010) found that the *adiponectin* −11377CG gene variant conferred a risk of T2DM in one Fujian Chinese population [8]. By contrast, Shi et al. (2007) did not find any association between *adiponectin* −11377CG gene polymorphism and T2DM in a northern Chinese population [9]. In this regard, Li et al (2010) found no correlation between them in the Yunnan Chinese population yet [10].

In the current study, a meta-analysis of 12 individual studies with a total of 6425 subjects (3237 with T2DM) was conducted to determine whether there was a relationship between *adiponectin* −11377CG gene polymorphism and T2DM in the Chinese population (Supplement S1).

## Materials and Methods

### Publication search and inclusion criteria

The words as “adiponectin,” “−11377,” “type 2 diabetes mellitus,” and “polymorphism” were used to search electronic databases, including PubMed, Embase, Web of Science, China National Knowledge Infrastructure, and China Biological Medicine Database. The last research was updated on January 26, 2013 with publication years ranging from 2007 to 2011.

The selected studies had to be in accordance with the following major criteria. a) The adiponectin −11377CG gene polymorphism and T2DM must be evaluated. b) The T2DM diagnosis criteria were derived from the American Diabetes Association fasting plasma criteria (2005). The fasting plasma glucose level was ≥7.0 mmol/L, and the 2 h plasma glucose of oral glucose tolerance test was ≥11.1 mmol/L. In addition, there must be no genetic relationships among participants in the individual studies. c) The individual studies should be case-control or cohort studies published in the official journals or peer-reviewed postgraduate dissertations. d) The study should be in agreement with the Hardy-Weinberg equilibrium (HWE).

### Data extraction

The data were abstracted according to a standard protocol. Three investigators conducted the meta-analysis; two of whom sought out parallel studies, and the third investigator served as the arbitrator to resolve the disagreement between the other two investigators. Studies that did not follow the inclusion criteria, those considered double publications, or those that provided inadequate data were excluded. If the same data appeared in different studies, the data were used only once. The abstracted data comprised the following items: the first author's name, publication year, region, number of genotypes, genotyping, study design, matching criteria, and total number of cases and controls.

### Statistical analyses

Six genetic models were used, including allelic (distribution of G allelic frequency of *adiponectin* −11377CG gene polymorphism), recessive (GG vs. CC+CG), dominant (CG+GG vs. CC), homozygous (GG vs. CC), heterozygous (CG vs. CC), and additive (G vs. C). The odds ratios (ORs) and their corresponding 95% confidence intervals (CIs) were used to compare the association between *adiponectin* −11377CG gene polymorphism and T2DM. Chi-square-based Q-tests were used to calculate the heterogeneity between the individual studies with significance set at the *P*<0.05 level [11]. The random-effect model was used to assess the pooled OR (DerSimonian and Laird method) if there was heterogeneity among the individual studies [12]. Otherwise, the fixed-effect model was used (the Mantel–Haenszel method) [13]. The pooled OR was determined through Z test with significance set at the *P*<0.05 level.

Fisher's exact test was used to evaluate the HWE, and significance was set at the *P*<0.05 level. The funnel plot was used to estimate the potential publication bias. Egger's linear regression test on the natural logarithm scale of the OR was used to assess the funnel plot asymmetry with significance set at the *P*<0.05 level [14]. STATA 11.0 software was used to perform the statistical analyses (StataCorp, College Station, TX, USA).

## Results

### Studies and populations

A total of twenty one studies were searched out, twelve of which met the inclusion criteria. Among the nine excluded studies, two papers were repeated publications, three papers were reviews, and four papers were not involved with *adiponectin* −11377CG or T2DM. No study was discarded for deviating from the HWE. The data were extracted from 3237 T2DM cases and 3188 controls (Table 1, Supplement S2) [7]–[10], [15]–[22]. The eleven study provinces included Heilongjiang, Fujian, Beijing, Qinghai, Shanxi, Sichuan, Shanghai, Liaoning, Hunan, Yunnan, and Guangxi. Two ethnicities, namely, Han and Hui, were included in the current meta-analysis.

**Table 1:** Characteristics of the investigated studies of the association of *adiponectin* −11377 CG gene polymorphism and type 2 diabetes mellitus (T2DM) in the Chinese population.

Author	Year	Ethnicity	Age(years old)		Gender(female/male)		BMI(kg/m^2^)		Region	T2DM			Control			sample size(T2DM/control)
			T2DM	Control	T2DM	Control	T2DM	Control		CC	CG	GG	CC	CG	GG	
Shi XH [9]	2007	Han	61..06±12.02^*^	47.52±7.43	222/126^*^	205/173	25.23±3.66^*^	23.86±2.97	Heilongjiang	171	151	26	180	173	25	348/378
Cai QY [15]	2008	Han	58.38±10.41	56.20±11.94	84/111	41/55	25.06±3.22	24.44±2.76	Fujian	115	67	13	56	36	4	195/96
Jia M [16]	2008	Han	61.00±14.89^*^	59.00±9.00	101/112	28/30	25.51±6.12	untested	Beijing	112	90	11	37	19	2	213/58
Wang Y [17]	2008	Hui	53.40±11.50^*^	48.20±12.00	68/54	52/52	22.80±3.60	22.70±3.50	Qinghai	54	53	15	54	47	3	122/104
Yang M [18]	2008	Han	50.00±9.00^*^	46.00±7.00	137/75	378/207	27.30±5.40^*^	23.90±3.40	Beijing	100	90	22	325	210	50	212/585
Ye F [7]	2008	Han	54.65±11.81^*^	57.93±12.13	84/120^*^	28/73	26.05±3.10^*^	24.88±2.91	Shanxi	108	78	18	53	38	10	204/101
Wang XZ19]	2009	Han	57.80±11.30	58.70±9.10	62/86	155/264	untested	untested	Sichuan	84	51	13	243	161	15	148/419
Wang YB [20]	2009	Han	64.90±10.60^*^	58.70±9.60	600/385^*^	686/312	25.56±2.42^*^	24.46±2.32	Shanghai	476	379	79	529	408	61	934/998
Zhao HY [21]	2009	Han	60.00±12.30^*^	62.00±10.40	82/90^*^	29/57	untested	24.50±3.60	Liaoning	130	30	12	64	18	4	172/86
Sun H [8]	2010	Han	49.00±11.00	47.00±11.00	117/138	53/67	25.00±3.00	24.00±4.00	Hunan	122	119	14	74	41	5	255/120
Li YP [10]	2011	Han	49.12±12.19^*^	36.64±14.14	81/121	84/59	24.52±3.26	21.45±3.42	Yunnan	110	75	17	68	65	10	202/143
Min Y [22]	2011	Han	55.80±13.28	54.98±14.04	97/135	40/60	24.88±2.88^*^	22.78±2.59	Guangxi	114	102	16	65	29	6	232/100

Abbreviations:

T2DM: type 2 diabetes mellitus; BMI: body mass index.

Allele-specific polymerase chain reaction (AS-PCR) was adopted in **Wang YB study**.

Polymerase chain reaction-restriction fragment length polymorphism (PCR-RFLP) adopted in all of the other studies.

Case-control study design has been adopted in all of the above studies.

### Pooled analyses

**Figure 1:**
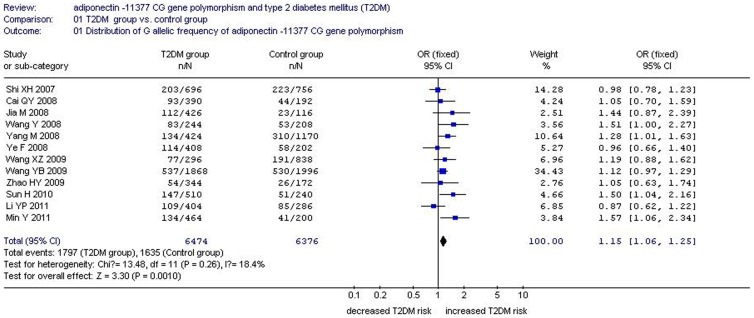
Forest plot of T2DM associated with *adiponectin* −11377 CG gene polymorphism under an allelic genetic model (distribution of G allelic frequency of *adiponectin* −11377 gene).

**Figure 2:**
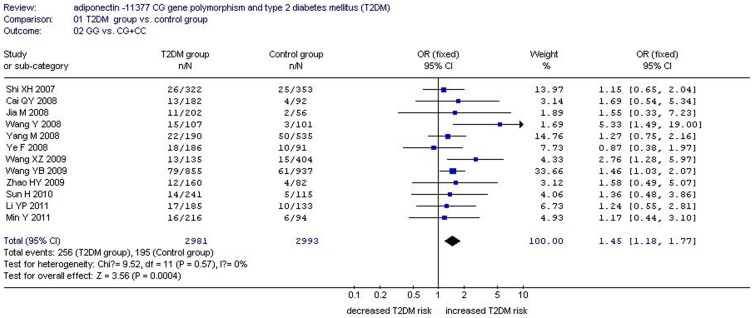
Forest plot of T2DM associated with *adiponectin* −11377 CG gene polymorphism under a recessive genetic model (GG vs. CG+CC).

**Figure 3:**
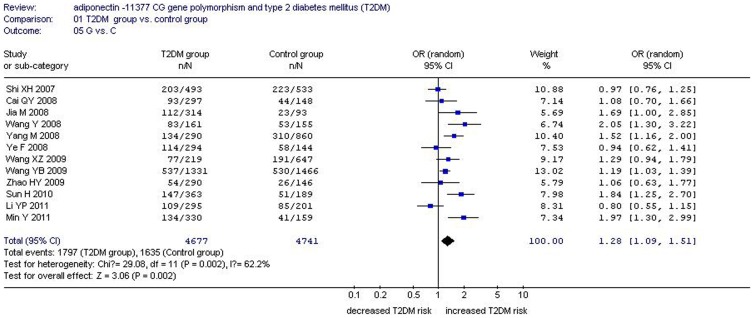
Forest plot of T2DM associated with *adiponectin* −11377 CG gene polymorphism under an additive genetic model (G vs. C).

**Figure 4:**
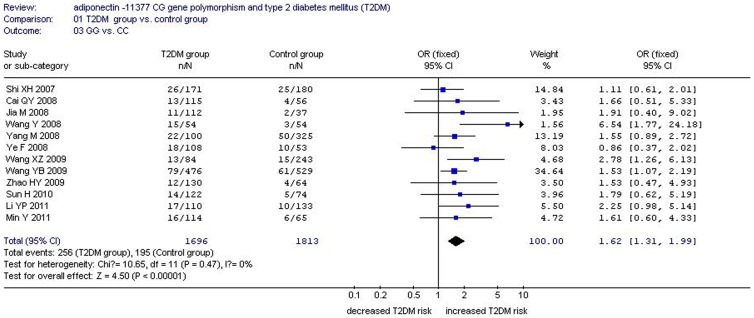
Forest plot of T2DM associated with *adiponectin* −11377 CG gene polymorphism under a homozygous genetic model (GG vs. CC).

**Figure 5:**
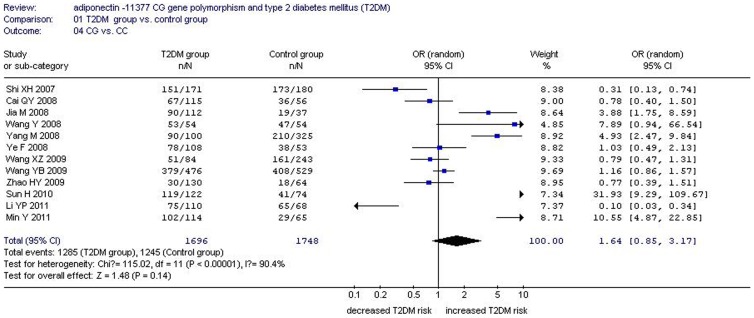
Forest plot of T2DM associated with *adiponectin* −11377 CG gene polymorphism under a heterozygous genetic model (CG vs. CC).

There was a significant relationship between *adiponectin* −11377CG gene polymorphism and T2DM under the allelic (OR: 1.150, 95% CI: 1.060–1.250, *P* = 0.001), recessive (OR: 1.450, 95% CI: 1.180–1.770, *P* = 0.0004), dominant (OR: 1.071, 95% CI: 1.013–1.131, *P* = 0.015), additive (OR: 1.280, 95% CI: 1.090–1.510, *P* = 0.002), and homozygous genetic models (OR: 1.620, 95% CI: 1.310–1.990, *P*<0.00001). No significant association was found between them under the heterozygous genetic model (OR: 1.640, 95% CI: 0.850–3.170, *P* = 0.140) (Table 2, Figures 1–5).

**Table 2:** Summary of meta-analysis of association of *adiponectin* −11377 CG gene polymorphism and type 2 diabetes mellitus (T2DM) in the Chinese population.

Genetic model	Pooled OR (95% CI)	P value	Study number	T2DM size	control size	*P*_heterogeneity(*I2%*)_
Allelic genetic model	1.150(1.060–1.250)	0.001*	12	3237	3188	0.26(18.4%)
Recessive genetic model	1.450(1.180–1.770)	0.0004*	12	3237	3188	0.48(0%)
Dominant genetic model	1.071(1.013–1.131)	0.015*	12	3237	3188	0.094 (37.2%)*
Additive genetic model	1.280(1.090–1.510)	0.002*	12	3237	3188	0.002 (62.2%)*
Subgroup 1: T1<205	1.140(0.880–1.430)	0.32	6	1043	949	0.04(56.9%)*
Subgroup 2: T1>205	1.420(1.140–1.750)	0.001*	6	2194	2039	0.008 (68.0%)*
homozygous genetic model	1.620(1.310–1.990)	<0.00001*	12	3237	3188	0.47(0%)
heterozygous genetic model	1.640(0.850–3.170)	0.140	12	3237	3188	<0.00001 (90.4%)*
Subgroup 1: T0<110	2.170(0.840–5.590)	0.11	6	1138	545	<0.00001 (87.5%)*
Subgroup 2: T0>110	1.260 (0.460–3.480)	0.65	6	2099	2643	<0.00001 (93.0%)*

* P<0.05.

Abbreviations:

T2DM: type 2 diabetes mellitus; CI: confidence interval; OR: odds ratio; T2D size: the total number of T2DM cases; control size: the total number of control group;

Allelic genetic model: G allele distribution frequency; recessive genetic model: GG vs. CG+CC; Dominant genetic model: CG+GG vs. CC; Additive genetic model: total G allele vs. total C allele; Homozygous genetic model: GG vs CC; heterozygous genetic mode: CG vs. CC.

T0: total number of control group; T1: total number of T2DM group.

Significant heterogeneity was observed under the additive (P_heterogeneity_ = 0.002, *I^2^* = 62.2%) and the heterozygous (P_heterogeneity_<0.00001, *I^2^* = 90.4%) genetic models. Meta-regression was performed subsequently to explore the heterogeneity source.

**Figure 6:**
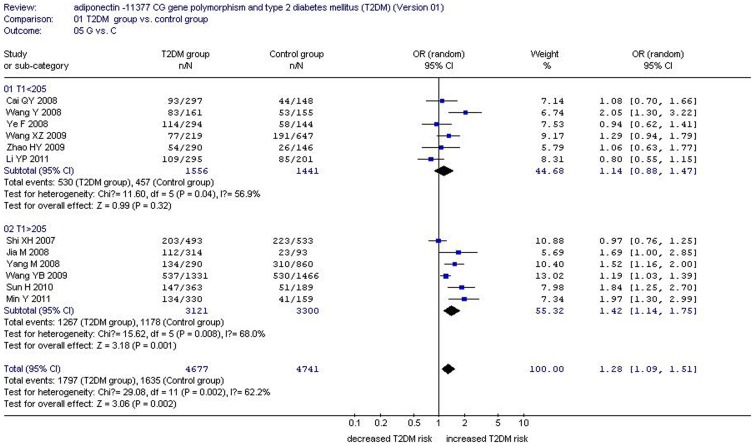
Forest plot of T2DM associated with *adiponectin* −11377 CG gene polymorphism under an additive genetic model stratified by T1 (G vs. C).

Under the additive genetic model, heterogeneity can be explained by the total number of the T2DM group (T1, *P* = 0.002), CC genotype number of control group (CC0, *P* = 0.009), and CG genotype number of the T2DM group (CG1, *P* = 0.086). According to T1, the entire population was also divided into two subgroups. The studies with T1<205 were encompassed in subgroup 1 and other studies with T1>205 were categorized into subgroup 2. In the subgroup analysis, significantly increased T1DM risk was only observed in subgroup 2 (OR: 1.420, 95% CI: 1.140–1.750, *P* = 0.001) and not in subgroup 1 (OR: 1.140, 95% CI: 0.880–1.430, *P* = 0.32). Heterogeneity was distinctly reduced, although it was still significant in both subgroups (subgroup 1: P_heterogeneity_ = 0.04, *I^2^* = 56.9%; subgroup 2: P_heterogeneity_ = 0.008, *I^2^* = 68.0%) (Tables 2–3, Figure 6).

**Table 3:** The meta-regression results among 12 studies under the additive genetic model for the association of *adiponectin* −11377 CG gene polymorphism and type 2 diabetes mellitus (T2DM) in the Chinese population.

	Coefficient	Standard Error	T value	P value	95% Confidence Interval
CC0	0.0034812	0.0009639	3.61	0.009*	0.0012018∼0.0057605
CG1	0.0212376	0.0043681	4.86	0.002*	0.0109086∼0.0315665
T1	−0.0088871	0.0018402	−4.83	0.002*	−0.0132383∼−0.0045358
_cons	2.193005	0.4318519	5.08	0.001	1.171837∼3.214172

* :P<0.05.

Coefficient: regression coefficient.

The regression coefficients are the estimated increase in the lnOR per unit increase in the covariates. cons: constant item.

T1: total number of T2DM group; CC0: CC genotype number of control group; CG1: CG genotype number of T2DM group sample size.

**Figure 7:**
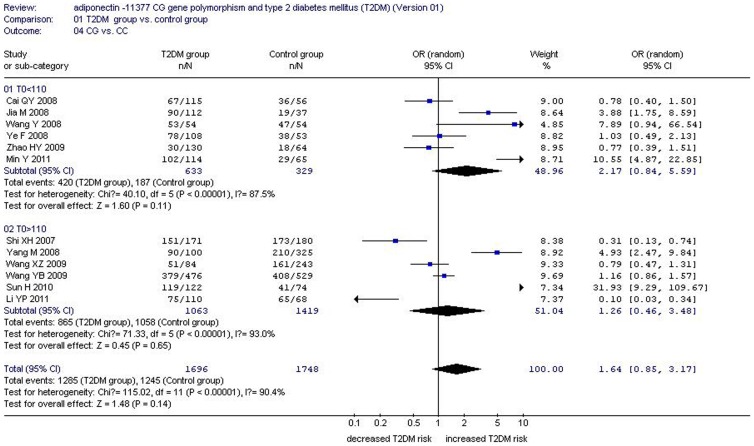
Forest plot of T2DM associated with *adiponectin* −11377 CG gene polymorphism under a heterozygous genetic model stratified by T1 (CG vs. CC).

Under the heterozygous genetic model, heterogeneity could be explained by T1 (*P* = 0.004), study region (*P* = 0.026), CG1 (*P* = 0.003), total number of control group (T0, *P* = 0.002), and CC0 (*P* = 0.002). According to T0, the studies with T0<110 were included in subgroup 1 and other studies with T0>110 were categorized into subgroup 2. In the subgroup analysis stratified by T0, the association between *adiponectin* −11377CG gene polymorphism and T2DM was strengthened but not significant in subgroup 1 (OR: 2.170, 95% CI: 0.840–5.590, *P* = 0.11) and in subgroup 2 (OR: 1.260, 95% CI: 0.460–3.480, *P* = 0.65). Meanwhile, heterogeneity was not weakened in both subgroups (subgroup 1: P_heterogeneity_<0.00001, *I^2^* = 87.5%; subgroup 1: P_heterogeneity_<0.00001, *I^2^* = 93.0%) (Table 2 and Table 4, Figure 7). Although heterogeneity still existed in both subgroups, the association between them was enhanced partly in subgroup 1, of which the OR was increased from 1.640 to 2.170.

**Table 4:** The meta-regression results among 12 studies under the heterozygous genetic model for the association of *adiponectin* −11377 CG gene polymorphism and type 2 diabetes mellitus (T2DM) in the Chinese population.

	Coefficient	Standard Error	T value	P value	95% Confidence Interval
T1	−0.031263	0.00522	−5.99	0.004*	−0.045756∼−0.0167701
Study region	1.418608	0.4104643	3.46	0.026*	0.2789769∼2.55824
CG1	0.1011914	0.015311	6.61	0.003*	0.0586813∼0.1437015
T0	−0.095547	0.0135318	−7.06	0.002*	−0.1331171∼−0.0579768
CC0	0.1653146	0.0213856	7.73	0.002*	0.1059387∼0.2246905
_cons	0.787407	1.382888	0.57	0.600	−3.052105∼4.626919

* :P<0.05.

Coefficient: regression coefficient.

The regression coefficients are the estimated increase in the lnOR per unit increase in the covariates. cons: constant item.

T1: total number of T2DM group; CG1: CG genotype number of T2DM group; T0: total number of control group; CC0: CC genotype number of control group;

### Bias diagnostics

**Figure 8:**
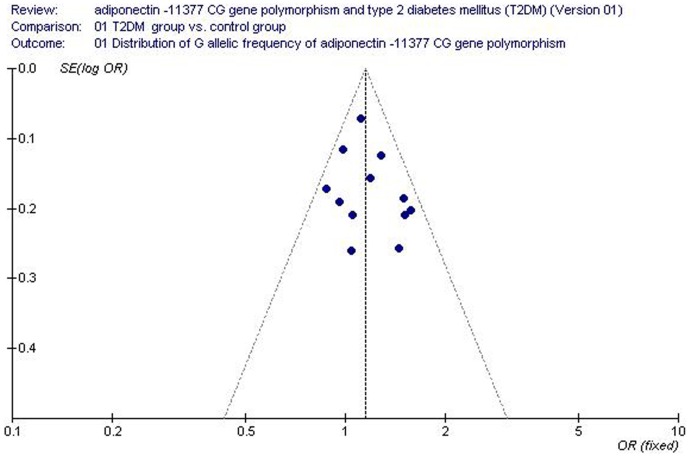
Funnel plot for studies of the association of T2DM associated with *adiponectin* −11377 CG gene polymorphism under an allelic genetic model (distribution of G allelic frequency of *adiponectin* −11377 gene). The horizontal and vertical axis correspond to the OR and confidence limits. OR: odds ratio; SE: standard error.

Funnel plot and Egger's test were used to assess the publication bias of the individual studies. No visual publication bias was observed in the funnel plot (Figure 8). No significant difference was detected in the Egger's test, which implied that there was no publication bias in the present meta-analysis (allelic genetic model, T = 1.01, *P* = 0.338).

## Discussion

The current meta-analysis showed a significant relationship between *adiponectin* −11377CG gene polymorphism and T2DM under the allelic (OR: 1.150), recessive (OR: 1.450), dominant (OR: 1.071), additive (OR: 1.280), and homozygous genetic models (OR: 1.620). No significant association was found between them under the heterozygous genetic model (OR: 1.640). Based on the current study indicating that *adiponectin* −11377 G allele might increase T2DM risk, it can be concluded that the G allele might confer T2DM susceptibility to the Chinese population.

Given that there was heterogeneity under the additive and heterozygous genetic models (P_heterogeneity_<0.05), meta-regression was performed to seek the heterogeneity source. In the subsequent heterogeneity source analysis, under the additive genetic model, the heterogeneity was found to be large and T1 partly explained the heterogeneity. With regards the heterozygous genetic model, T0 was the main heterogeneity source (*P*<0.05). Moreover, T1 or T0 should be better matched in the individual studies to decrease the heterogeneity.

In the current meta-analysis, most of the individual studies were on the Han ethnicity, and only one study focused on the Hui ethnic group of Qinghai Province [17]. The Hui ethnicity research results demonstrated that the G allele frequency of *adiponectin* −11377CG gene polymorphism was 27.93%, which was either similar to the 29.6% mutation rate in the Chinese northern Han population [9] or close to that in the Shanxi Han population at 27.9% [7]. Thus, there was no evidence of a significant difference in the *adiponectin* −11377CG gene mutation rate among different regions and ethnicities. As such, the genetic diversity did not exist in the different regions and ethnicities of the Chinese population.

Adipocytes have various endocrine, paracrine, and autocrine functions. Adiponectin is a cytokine specifically secreted by adipose tissue. Meijer et al. found extremely low adiponectin expression of preadipocytes compared with adipocytes. Thus, the adiponectin is considered as a marker for adipocytes differentiation [23]. Adiponectin has a number of key physiological functions, and most of them are mediated by the activation of AMP-activated protein [24]. Adiponectin can improve the glycolipid metabolism and IR; it can also restrain the adhesion molecule expression in the human aorta endothelial cells and exert the anti-atherosclerosis effects. Adiponectin can regulate the inflammatory response by inhibiting the pre-macrophage growth and mature macrophage function. Chen et al. investigated the relationships between the levels of inflammation, adiponectin, and oxidative stress in subjects with metabolic syndrome (MS), and found that a higher level of hs-CRP (≥1.00 mg/L) or IL-6 (≥1.50 pg/mL) or a lower level of adiponectin (<7.90 µg/mL) were associated with a significantly greater risk of MS. They concluded that a higher inflammation status was significantly correlated with a decrease in the adiponectin level and an increase in the risk of MS [25]. The current study verified again that adiponectin had an anti-inflammation effect.

Although the exact mechanism of the association of *adiponectin* −11377CG gene polymorphism and T2DM has not been clarified, decreased or deficient serum adiponectin levels presumably contributed to the T2DM risk. Adiponectin is a significant adipokine, which is only expressed and secreted by the adipose tissue [26]. Adiponectin has anti-inflammatory and anti-atherosclerotic properties, and as such, it can increase insulin sensitivity. In most cases, the adiponectin levels are significantly decreased in patients with obesity, IR, T2DM, and cardiovascular diseases [4]. The −11377CG gene locus, rs266729, is in the upstream of the transcription start point, and the −11377CG CG variant alters some transcription regulation elements as well as influences the adiponectin secretion. Vasseur et al. reported that a nucleotide sequence [tcctgc] was next to the −11377 position, which was similar to an enhanced element of the epidermal growth factor receptor (EGFR). They speculated that this nucleotide sequence might indirectly influence the adiponectin gene expression and lead to T2DM [27]. Meanwhile, Zhang et al. found that −11377 CG loci was in the SP1 binding site, and when the C allele was substituted by the G allele, the SP1 binding site disappeared, thus contributing to the decreased plasma adiponectin level [28]. Sun et al. found that the −11377G allele was distinctly associated with low plasma adiponectin level and IR, thus resulting in T2DM risk [8].

Recent clinical studies have reported that low-grade inflammation has a significant function in the T2DM [24], [29]. Tian et al. found that oral treatment with γ-aminobutyric acid improved glucose tolerance and insulin sensitivity by inhibiting inflammation in mice fed with a high-fat diet [30]. Given that adiponectin could inhibit the inflammatory reaction, the lower plasma adiponectin level resulting from the −11377 CG mutation aggravated the inflammation level, leading to the T2DM progress.

Han et al. also conducted a meta-analysis of the relationship between *adiponectin* −11377CG gene polymorphism and T2DM, and concluded that the *adiponectin* −11377 G allele was a risk factor for T2DM [31]. Despite the similar conclusion obtained from the current meta-analysis, there are still certain defects in their work. For example, although gene distribution varies among different populations, Han et al. combined the white, black, Japanese, and Chinese populations to explore the association between *adiponectin* −11377CG gene polymorphism and T2DM. In addition, they did not conduct subgroup analysis stratified by population. Regarding the Chinese population studies, only five were included, and as such, the number of studies was inadequate. In the current meta-analysis, twelve studies were searched, but their literature retrieval was not completed. Additionally, the study conducted by Tso listed 48 references but it was not suitable for inclusion in the meta-analysis, because the T2DM group included the impaired glucose tolerance patients. In this sense, the work of Han et al. seemed to lack objectivity and credibility.

There are certain limitations in the current meta-analysis. Large-scale studies on the association of T2DM with *adiponectin* −11377CG gene polymorphism remain insufficient. The adiponectin expression level was influenced not only by the *adiponectin* −11377CG gene polymorphism, but also by other hereditary and environmental factors. Given that T2DM is a multigenic hereditary disease, *adiponectin* −11377CG gene polymorphism can be associated with the gene linkage disequilibrium as *adiponectin* −11391GA, +45TG, and +276GT gene polymorphisms, which can influence T2DM development [31].

In conclusion, *adiponectin* −11377CG gene polymorphism was obviously associated with T2DM susceptibility in the Chinese population. People with the G allele are predisposed to T2DM. This conclusion contributes to the formulation of more effective individual T2DM therapy strategies. In consideration of the aforementioned limitations, more large-scale studies are necessary to validate the significance of our findings.

## Supporting Information

Supplement S1 **PRISMA 2009 Checklist.**
(DOC)

Supplement S2 **PRISMA 2009 Flow Diagram.**
(DOC)
