# Supplementary material for: Adiponectin-11377CG Gene Polymorphism and Type 2 Diabetes Mellitus in the Chinese Population: A Meta-Analysis of 6425 Subjects
Source: PLoS One. 2013 Apr 9;8(4):e61153. doi: 10.1371/journal.pone.0061153 (PMC3621968; doi:10.1371/journal.pone.0061153)
Supplement: Supplement S2 — PRISMA 2009 Flow Diagram. (DOC) [file pone.0061153.s002.doc]

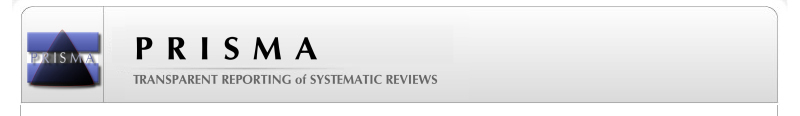
**PRISMA 2009 Flow Diagram**

**Screening**

**Included**

**Eligibility**

**Identification**

Records identified through database searching
(n =21 )

Additional records identified through other sources
(n =0 )

Records after duplicates removed
(n =19)

Records screened
(n =16 )

Records excluded for review characteristic
(n =3 )

Full-text articles assessed for eligibility
(n =16)

Full-text articles excluded for deviation from HWE (n =0 )

Studies assessed for eligibility
(n =12)
(n =9)

Records excluded for no association with adiponectin -11377CG or T2DM

(n =4)

Records excluded for duplicated publication (n = 2)
